# Untargeted characterisation of dissolved organic matter contributions to rivers from anthropogenic point sources using direct‐infusion and high‐performance liquid chromatography/Orbitrap mass spectrometry

**DOI:** 10.1002/rcm.8618

**Published:** 2020-02-13

**Authors:** Jonathan A. Pemberton, Charlotte E.M. Lloyd, Christopher J. Arthur, Penny J. Johnes, Michael Dickinson, Adrian J. Charlton, Richard P. Evershed

**Affiliations:** ^1^ Organic Geochemistry Unit, School of Chemistry University of Bristol Cantock's Close Bristol BS8 1TS UK; ^2^ School of Chemistry University of Bristol Cantock's Close Bristol BS8 1TS UK; ^3^ School of Geographical Sciences University of Bristol University Road Bristol BS8 1SS UK; ^4^ Fera Science Ltd Sand Hutton York YO41 1LZ UK

## Abstract

**Rationale:**

Anthropogenic organic inputs to freshwaters can exert detrimental effects on aquatic ecosystems, raising growing concern for both environmental conservation and water security. Current regulation by the EU water framework directive (European Union, 2000/60/EC) relates to organic pollution by monitoring selected micropollutants; however, aquatic ecosystem responses require a comprehensive understanding of dissolved organic matter (DOM) composition. The introduction of high‐resolution mass spectrometry (HRMS) is set to greatly increase our understanding of the composition of DOM of both natural and anthropogenic origin derived from diffuse and point sources.

**Methods:**

DOM was extracted from riverine and treated sewage effluent using solid‐phase extraction (SPE) and analysed using dissolved organic carbon analysis, direct‐infusion high‐resolution mass spectrometry (DI‐HRMS) and high‐performance liquid chromatography (HPLC)/HRMS. The data obtained were analysed using univariate and multivariate statistics to demonstrate differences in background DOM, anthropogenic inputs and in‐river mixing. Compound identifications were achieved based on MS^2^ spectra searched against on‐line databases.

**Results:**

DI‐HRMS spectra showed the highly complex nature of all DOM SPE extracts. Classification and visualisation of extracts containing many thousands of individual compounds were achieved using principal component analysis (PCA) and hierarchical cluster analysis. Kruskal‐Wallis analyses highlighted significant discriminating ions originating from the sewage treatment works for more in‐depth investigation by HPLC/HRMS. The generation of MS^2^ spectra in HPLC/HRMS provided the basis for identification of anthropogenic compounds including; pharmaceuticals, illicit drugs, metabolites and oligomers, although many thousands of compounds remain unidentified.

**Conclusions:**

This new approach enables comprehensive analysis of DOM in extracts without any preconceived ideas of the compounds which may be present. This approach has the potential to be used as a high throughput, qualitative, screening method to determine if the composition of point sources differs from that of the receiving water bodies, providing a new approach to the identification of hitherto unrecognised organic contribution to water bodies.

## 1 INTRODUCTION

Fresh surface water is a fundamental resource not only for drinking water and irrigation, but also for supporting terrestrial and aquatic ecosystems.^1^, ^2^ Dissolved organic matter (DOM) is ubiquitous to all aquatic systems and is an extremely complex mixture of organic compounds although its composition has remained intractable due to the lack of suitable analytical methods.^3^ DOM has been asserted to be a nutrient for autotrophs.^2^, ^3^ The range of compounds comprising DOM includes compounds generated naturally and through anthropogenic activities, and can include potentially toxic micropollutants which attract much attention in water quality legislation as they have been shown to have adverse impacts upon organisms within the aquatic ecosystems.^4^, ^5^, ^6^ Despite individual anthropogenically derived compounds being at low concentration, the chronic exposure of stream biota to these compounds has been shown to have a wide range of acute ecotoxicological and chronic adverse effects on organisms.^4^, ^5^, ^6^, ^7^ These include the disruption of reproduction,^8^, ^9^ a reduction in biodiversity,^10^ and dysmorphia in the maturation of organisms.^11^ Furthermore, different compounds which affect organisms in a similar way can work synergistically amplifying the impact.^7^, ^12^ Regulations only cover a very minor proportion of the commonly identified micropollutants, and many others almost certainly remain to be discovered.

Micropollutants have been identified in different discharges including sewage treatment works.^13^, ^14^ Sewage treatment works have been found to be a major gateway for the release of pharmaceuticals,^15^ personal care products,^16^ and plasticisers^17^ into the environment. The concentration and presence/absence of target compounds across different sewage treatment works have been found to vary between different sites and over time.^13^, ^15^, ^16^, ^18^, ^19^ With over 9000 sewage treatment works in the UK and numerous other point sources, the identification of potentially ecotoxicological compounds remains a challenge. Without identifying these micropollutants; the determination of ecotoxicity, effective mitigation solutions and environmental monitoring cannot be carried out.

The most common approach to the determination of organic compounds in both wastewater and the natural aquatic environment ecosystem is targeted analysis using mass spectrometry (MS) approaches focusing on known or suspected compounds.^4^, ^20^ Optimised extraction methods are used to isolate and concentrate the target analytes with subsequent interrogation involving gas chromatography (GC)^21^, ^22^ or high‐performance liquid chromatography (HPLC)^13^, ^23^ linked to MS. Targeted studies have largely focused on pharmaceuticals,^15^, ^24^ personal care products,^16^, ^25^ and pesticides,^21^, ^26^ with their concentrations or load in the riverine environment being used to assess the effectiveness of sewage treatment and local sources.^13^, ^15^, ^27^ The obvious limitation of targeted analysis is that it requires a predetermined list of known compounds. Targeted analysis will only determine the selected compounds and exclude other compounds originating from a point source or the environment. The use of electrospray ionisation (ESI) and high‐resolution mass spectrometry (HRMS) has revolutionised the analysis of complex mixtures of water‐soluble compounds, such as DOM, allowing the exact masses of individual molecules to be determined.^28^, ^29^ The ionisation of intact molecules and their mass analysis using instruments with high resolving power and high mass accuracy mean that each ion in a spectrum potentially corresponds to a unique compound (taking account of adducts and isotopes). Application of this approach has revealed the extraordinary complexity and heterogeneity of DOM in the natural environment, as evidenced by the DI‐HRMS spectra containing many thousands of resolved ions.^30^, ^31^

One of the major challenges of utilising these HR mass spectra of DOM lies in the interrogation of the data. Attempts have been made to assign formulae to the observed ions in the spectra, using rule‐based calculations.^32^, ^33^, ^34^ All studies include carbon, oxygen, nitrogen and hydrogen; however, the inclusion of heteroatoms, e.g. P, Cl and S, varies between studies.^30^, ^35^, ^36^ Increasing the number of heteroatoms results in an exponential increase in the number of possible formulae for a single ion, resulting in a high level of uncertainty and false positives.^33^ Isotopes and adducts, i.e. [M + Na]^+^, [M + K]^+^, [M + Cl]^−^, will be present in all DI‐HRMS spectra, but are rarely accounted for. Hence, despite the high mass resolution attainable using modern Fourier‐transform ion cyclotron resonance (FTICR) or Orbitrap™ MS instruments, the exceptional complexity of the mass spectra obtained largely defies conventional approaches to handling these unusual data sets.

An alternative approach is to move toward data visualisation rather than more conventional peak identification approaches. One such approach is the use of van Krevelen diagrams. Such diagrams use the ratios of carbon:hydrogen and carbon:oxygen of the formulae assigned to ions as a basis for the comparison of DOM in water extracts.^37^, ^38^, ^39^, ^40^, ^41^ These elemental ratios of formulae are used to classify ions to a compound class.^30^, ^31^, ^40^, ^41^ However, the interpretation of a van Krevelen diagram relies on the correct assignment of formulae, including appropriate numbers of heteroatoms. Incorrect assignments will lead to the inaccurate interpretations of differences in the composition of DOM extracts. Furthermore, a single ion in a DI‐HRMS spectrum may be the result of multiple isomers and, therefore, the full complexity is not revealed. In addition, the correct classification using a van Krevelen diagram of a compound class for one isomer may be incorrect for another isomer with the same formula. Despite this van Krevelen diagrams have found utility in visualising differences in composition of DOM extracts from different aquatic systems, addressing a range of questions relating to DOM source and variability between ecosystems, e.g. differences between water bodies in different geographical locations.^38^, ^42^, ^43^

While van Krevelen diagrams have proved useful for visualising differences between DOM extract chemistries, the approach is non‐statistical and is rather restricted in truly exploiting the full complexity of the data, e.g. ion intensities and molecular species of unassigned formulae. An alternative, but still less widely applied approach, is multivariate statistics, in particular principal component analysis (PCA) of DI‐HRMS spectra. The latter has been used to determine and visualise differences between the composition of DOM extracts from different solid‐phase extraction (SPE) methods^44^ and different water bodies within the same pristine catchment.^42^ PCA requires only the detected ions and their intensities in different DI‐HRMS spectra to determine if extracts are different. However, this approach has not been applied to point sources in comparison with their receiving environment.

Herein, we address the challenge of how to deal with the question of the complexity of riverine DOM analysis by HRMS. We have taken a comprehensive approach in order to retain a broad view of DOM composition and developed a method for data reduction based on a difference algorithm to highlight complex anthropogenic DOM contributions against a natural or semi‐natural DOM background. To achieve this, we first recorded DI‐HRMS spectra of DOM recovered by SPE, then used PCA as a rapid qualitative screening method to determine if differences exist between DOM extracts of point sources and the receiving aquatic environment. Following this the difference algorithm, employing univariate statistics (Kruskal‐Wallis analysis) was applied to allow the anthropogenic point source components to be identified in DI‐HRMS spectra. Heatmaps and hierarchical cluster analysis were then used as data visualisation tools, which allowed compositional differences to be recognised. The anthropogenic components highlighted through the untargeted difference analysis formed the basis for structural identification of specific molecular species by HPLC/HRMS/MS.

## 2 EXPERIMENTAL

### 2.1 Sampling

The sewage treatment works (at 51° 21′ 21.8052” N, 2° 37′ 2.262” W) is situated on the River Chew in Somerset, UK, which drains North West from its source at Chewton Mendip to the sewage works, and then North East to its confluence with the Bristol Avon at Keynsham (at 51° 25′ 7.7196” N, 2° 29′ 32.118” W). It is located downstream of Chew Valley Lake, a significant reservoir supplying water to the city of Bristol, UK. This site was selected to test and develop this method as it was close enough to the University of Bristol to allow rapid stabilisation of samples in cold storage following collection in the field (details of which are given below). The treatment methods used at the sewage treatment works include: (i) preliminary solid removal of large particulates, (ii) primary settling to further allow smaller particulates to flocculate and settle out of the water, and (iii) secondary treatment using trickle filter beds to biologically break‐down organic matter. The sewage treatment works has a tertiary treatment which includes phosphorus stripping. The final treated effluent is discharged into the river downstream of the reservoir.

Three comparative water samples (5.25 L) were collected in amber glass bottles. The first was taken *ca* 60 m upstream of the sewage treatment outfall. The second was taken directly from the discharging sewage outfall and the third *ca* 50 m downstream. Five procedural controls of HPLC grade water (1 L, Fisher Scientific, Loughborough, UK) were extracted with the water samples collected. The water was divided into 1‐L aliquots, which were vacuum filtered using an all glass filter apparatus (47 mm, Merck Millipore, Feltham, UK) through glass fibre filters (0.5 μm, 47 mm, Advantec, Cole‐Palmer, Hanwell, UK) within 24 h of collection. Both the filter and the filtration apparatus were pre‐combusted before use (450°C, 4 h). An additional 20 mL of each water sample was filtered using the same apparatus and retained to determine the concentration of dissolved organic carbon (DOC). The filtered water samples (1 L) were acidified to pH 2 using hydrochloric acid (30%, TraceSelect, Sigma–Aldrich, Gillingham, UK) and extracted using Oasis Hydrophilic–Lipophilic Balance (HLB) SPE cartridges (400 mg bed mass, 60 μm particle size, Waters Ltd, Elstree, UK). The cartridges were conditioned using HPLC grade methanol (3 mL, Rathburn Chemicals Ltd, Walkerburn, UK) and HPLC grade water (3 mL) before the acidified filtered water (1 L) was extracted. After extraction, the cartridges were rinsed with acidified HPLC grade water (3 mL, Fisher Scientific) and dried under vacuum for 30 min. The extracts were eluted from the SPE cartridges with HPLC grade methanol (6 x 1 mL) and dried under a steady stream of nitrogen. Dried extracts were dissolved in a mixture of HPLC grade methanol/water (1:1, v/v, 1 mL).

An aliquot of each extract (100 μL) was mixed to create a pooled quality control (QC) and an aliquot of each extract (50 μL) was removed and dried under a steady stream of nitrogen for DOC analysis. The pooled QC and all extracts were then stored at −85°C until required for analysis.

### 2.2 DOC analysis

The dried 50‐μL aliquots of the extracts were dissolved in MilliQ water (20 mL, Merck Millipore) before DOC analysis. Filtered water samples were analysed directly. All analyses were carried out using a TOC‐L analyser (Shimadzu, Milton Keynes, UK) using the non‐purgeable organic carbon (NPOC) method recommended by the manufacturer for the analysis of environmental water samples. The results for the mean of three to five injections of 150 μL, where the coefficient of variance for replicate injections was <2%, are presented in Table 1.

**Table 1:** DOC of the filtered water, concentration of organic carbon extracted using SPE and the efficiency of the extraction procedure

	DOC filtered water (mg C L^−1^)	DOC concentration of extracts (mg C L^−1^)	Extraction efficiency (%)
Upstream	3.39	1.50 ± 0.04	42.11 ± 0.5
Sewage outfall	3.19	1.29 ± 0.05	40.49 ± 1.7
Downstream	3.45	1.40 ± 0.06	40.34 ± 0.8
Blank	N/A	0.18 ± 0.02	N/A

### 2.3 DI‐HRMS analysis

DI‐HRMS spectra were recorded in positive ion mode using an Orbitrap™ Elite Hybrid Ion Trap‐Orbitrap™ mass spectrometer (Thermo Scientific, Hemel Hempstead, UK) with a heated electrospray ionisation (HESI) source. The instrument was calibrated using Thermo Scientific Pierce LTQ ESI Positive Ion Calibration Solution. The instrument had a mass error of 3.2 ppm and resolution of m/Δm 197,389 at *m/z* 524.257, and, upon tuning, the S‐lens radio frequency level was 61.81%. Extracts were directly infused at a rate of 5 μL min^−1^ into the HESI source. The source voltage was set to 3.0 kV, the sheath gas (nitrogen) flow rate to 10 arbitrary units (arb), the auxiliary gas (nitrogen) flow rate to 5 arb, the sweep gas (nitrogen) flow rate to 5 arb, and the capillary temperature to 275°C. The mass spectrometer was set to acquire in the mass range of *m/z* 150 to 2000 for 100 scans, and the ions detected were recorded in profile using the nominal resolving power “240,000”. The maximum injection time was set at 200 ms and the automatic gain control (AGC) target was set to 1,000,000. The total ion chromatogram (TIC) was assessed for any losses in signal during analysis. Extracts were analysed in random order. The mixed QC and calibration solutions were analysed after every five extracts, and the mass drift was 1.8 ppm over all analyses.

### 2.4 HPLC/HRMS and HPLC/HRMS/MS analysis

The SPE extracts (10 μL) were analysed by HPLC/HRMS using a Dionex Ultimate HPLC system (Thermo Scientific) coupled to an Orbitrap™ Elite Hybrid Ion Trap‐Orbitrap™ mass spectrometer with a HESI source. Chromatographic separation used an ACE UltraCore Super C_18_ column (150 x 2.1 mm i.d., 25 Å particle size; Hichrom, Reading, UK). The column was kept at a constant temperature of 50°C. The gradient program used HPLC grade water as mobile phase A and HPLC grade acetonitrile (Fisher Scientific) as mobile phase B, both with 0.1% formic acid (Fisher Scientific) as a modifier. The flow rate was kept constant at 350 μL min^−1^. The gradient program was as follows: 5% B for 1 min, 5% to 95% linear gradient for 30 min and 95% held for 5 min before returning to 5% in 1 min and remaining at 5% for 4 min. All spectra were recorded using the nominal resolving power at “120,000” in positive ion mode for the mass range *m/z* 150 to 2000 in centroid mode and the AGC target was set to 1,000,000. The source voltage was set to 3.5 kV, the source temperature to 80°C, the sheath gas (nitrogen) flow rate to 30 arbitrary units (arb), the auxiliary gas (nitrogen) flow rate to 10 arb, the sweep gas (nitrogen) flow rate to 10 arb, and the capillary temperature to 275°C. Between each analysis a solvent blank of HPLC water was run to ensure that there was no carry over between samples.

The data‐dependent acquisition (DDA) method was used for the acquisition of MS^2^ spectra for a target mass list of ions and their retention times. The HPLC method and source settings were consistent between the HPLC/HESI‐HRMS and HPLC/HESI‐HRMS/MS runs. Ions detected in the HPLC/MS spectra within 10 ppm of the *m/z* values of ions determined to be significant from the Kruskal‐Wallis analysis were compiled into a target mass list of *m/z* values and retention times. A stepwise method consisting of seven scan events was used. A full scan event was recorded using the nominal resolving power at “120,000” to identify the presence of a target mass ion. If a target mass ion was detected within the retention time range of 30 s, a series of six MS^2^ scans was recorded using the the Orbitrap™ with a nominal resolving power at “7000” at different collision‐induced dissociation (CID) energies of 10, 20, 30, 40, 50 and 60 eV. The same target ion could be recorded twice before it was excluded for 30 s.

### 2.5 Data processing

DI‐HESI‐HRMS files were converted from Thermo.raw to. mzML using MSConvert. All 100 scans were merged using an openMS spectramerger module in KNIME.^45^, ^46^ This was done because the XCMS package for the peak picking of DI‐HRMS expects a single mass spectrum. Ion picking and alignment were performed using the XCMS package (v 1.52.0) in R (v 3.4.0) to create a data matrix of ion intensities aligned by mass.^47^ The changes in the mass accuracy across the analytical run were assessed using the accurate mass of standard ions, and ions were aligned using a mass tolerance of 5 ppm. The ion had to be present in three of the five replicate DI‐HRMS analyses.

Files from the HPLC/MS analysis were converted from Thermo.raw to .mzML using MSConvert.^48^ Peak picking and alignment were performed using the XCMS (v 1.52.0) package in R (v 3.4.0) to create a data matrix of sample intensities aligned by mass and retention time.^47^, ^49^, ^50^ The method used for peak picking was the centWave algorithm which is recommended for peak picking and alignment of HPLC/HRMS data. Peaks were picked above a signal‐to‐noise (*s/n*) ratio of 10; the mass tolerance allowed was 10 ppm with a retention time tolerance range of 15 to 60 s. The peaks were then aligned across samples if the mass was within 0.002 *m/z* units and the retention times overlapped by 10 s.

HPLC/MS/MS files were converted from Thermo.raw to .mzML using MSConvert.^48^ Peak picking was performed using the XCMS (v 1.52.0) package in R (v 3.4.0).^47^, ^49^, ^50^ A data matrix of product ions and intensities was created corresponding to a specific precursor ion mass, retention time and the fragmentation energy. Product ion spectra were compared with spectra in two databases ‐ mzCloud and MassBank.

### 2.6 Statistical analyses and visualisation methods

All calculations and visualisations of the statistical analyses of the DI‐HRMS spectra were carried out using Mass Profiler Professional (Agilent Technologies Ltd, Abingdon, UK). The intensity of the ions was transformed using the log_2_ scale. The position and clustering of the mixed QC in the PCA were used to determine if there were any changes caused by analytical variance or data processing. Once this was shown to be minimal, the QC and blank data were removed and PCA and hierarchical cluster analyses were performed to determine the differences between the sample groups. Heat maps are generated automatically as part of the hierarchical cluster analysis and to visualise the difference in ion intensities between the mass spectra of the extracts. Kruskal‐Wallis analysis was then used to compare the sewage effluent and upstream DOM composition based on ion distributions and intensities to determine statistically significant ions (based on p values), which vary between the mass spectra. Ions with a p value <0.005, and that were found to increase in intensity when comparing the upstream and sewage outfall DI‐HRMS spectra, were compiled into a target list for further investigation using HPLC/HRMS (described above).

## 3 RESULTS AND DISCUSSION

The analytical approach described above aimed to identify the complex array of anthropogenic compounds discharged in treated sewage DOM against a background of riverine DOM. One of our primary objectives was to retain a comprehensive overview of the DOM composition in order that contributions that would be missed in targeted analyses can be routinely detected. This relates to our wider objective of developing an holistic understanding of the role of DOM in driving aquatic ecosystem ecology, rather than the more common goal of targeted analyses for the regulation of priority pollutants. The approach used proceeds in three phases: (i) DI‐HRMS analysis of water samples to identify the ions derived from the sewage effluent DOM against the background of natural riverine DOM, (ii) application of statistical methods to allow significant compositional differences to be determined and visualised diagrammatically, and (iii) use of HPLC/HRMS to further explore the complexity of DOM to identify individual molecular species through MS^2^ spectra.

### 3.1 DOC analyses of the DOM and SPE extracts

The DOC concentrations of the filtered water collected from each sampling site, the concentration of organic carbon recovered by SPE and, hence, the extraction efficiency of the SPE process, are shown in Table 1. These data reveal little difference in the DOC concentrations and the SPE extracts of the water samples from the sewage outfall and the river. The DOC concentrations were similar for all samples at *ca* 3 mg C L^−1^, sitting within the range of variation previously reported for UK rivers, including in this study, which ranged from 0.76 mg C L^−1^ in chalk catchments to >26 mg C L^−1^ in peat catchments.^51^ These data emphasise the ineffectiveness of DOC concentrations in revealing differences in the composition of the DOM pool, where markedly different compound mixes can share a similar DOC concentration. The DOC determinations do, however, provide a useful means of assessing the SPE recovery efficiencies of DOM from all three water samples, i.e. *ca* 40%, which is typical of the recoveries recorded for the HLB phase in other studies.^44^, ^52^ It should be noted that this SPE phase was chosen as it has been widely used in targeted^13^, ^15^, ^53^ and untargeted analyses,^54^, ^55^ and passive sampling.^56^, ^57^ The similarities in DOC concentration and extraction efficiencies emphasise the need to explore alternative, i.e. molecular, approaches, to gain an in‐depth understanding of the composition characteristics and potential ecological impacts in relation to DOM source.

### 3.2 DI‐HRMS analysis of SPE extracts

**Figure 1:**
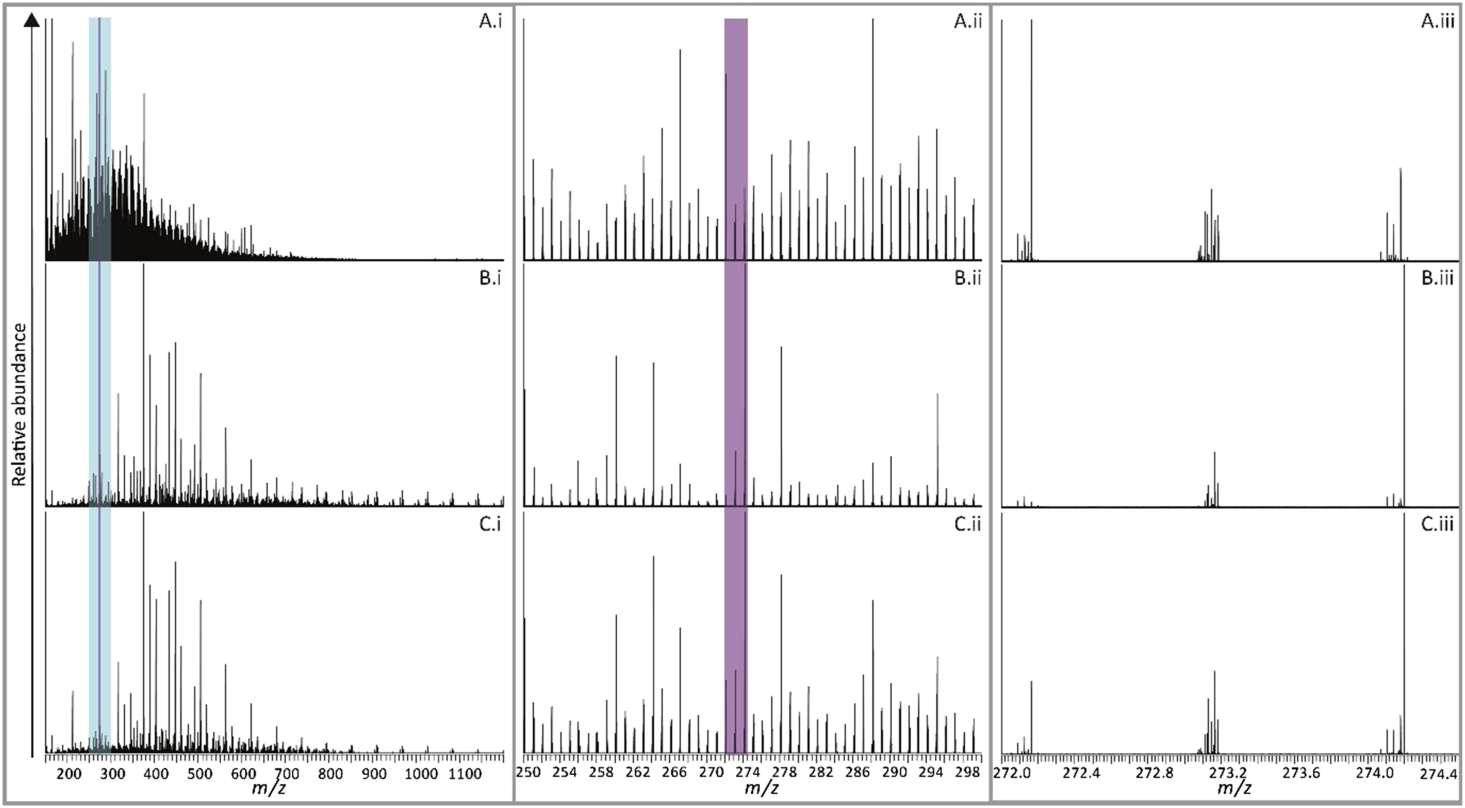
DI‐HRMS spectra of A, the upstream, B, the sewage outfall, and C, the downstream SPE extracts displaying the mass ranges *m/z* 150 to 1200 (A.i, B.i, and C.i), *m/z* 250 to 300 (A.ii, B.ii, and C.ii), and *m/z* 272.0 to 274.4 (a.iii, B.iii, and C.iii)

The DI‐HRMS spectra of the upstream, sewage outfall and downstream extracts are shown in Figure 1. The spectra of all three DOM SPE extracts show the remarkable complexity of the composition of both the riverine and the sewage effluent extracts. The full mass range spectra show clear differences between sources. The upstream spectrum (Figure 1(A.i)) shows a similar character to SPE extracts of DOM from other studies of riverine DOM.^30^, ^58^ The spectrum shows an extremely high density of ions in the range *m/z* 150 to 750, maximising at *m/z* 288.1956. In contrast, the sewage effluent and downstream extracts differ markedly in composition from the upstream extract. These two extracts are characterised by a prominent series of ions extending well beyond *m/z* 1000 (Figures 1(B.i and C.i)). The differences in composition between these two extracts and the upstream DOM reveal a very significant contribution from the sewage works to the riverine DOM, suggesting overprinting of the river background DOM by the anthropogenic contribution. Preliminary assessment of this contribution reveals a prominent series of ions differing by 58 *m/z* units with the intensities describing a slightly skewed normal distribution, suggestive of the presence of a polymer, or perhaps more correctly a mixture of oligomers.

Eighteen oligomeric series containing 156 ions were identified with a mass difference of 58.0419 ± 0.005 which indicates a structural motif of [CH_2_CH_2_CH_2_O], consistent with the presence of oligomers of the synthetic industrial polymer polypropylene glycol (PPG). Further investigation of the DI‐HRMS spectra points to the presence of a number of variants of the PPG series, which will be discussed in detail below. Based on this preliminary assessment alone, the sewage outfall DOM has clearly profoundly affected the composition of the river DOM.

Figures 1(ii and iii) show examples of two selected mass range windows, i.e. *m/z* 250 to 300 (blue highlighted mass window in the Figure 1(i) spectra) and *m/z* 272.0 to 274.0 (purple highlighted mass window in the Figures 1(i and ii) spectra), of the full DI‐HRMS spectra. These spectra illustrate the exquisite compositional detail revealed through use of high mass resolution (*m/Δm* = “240,000”), in particular, differences in composition between the upstream, sewage works discharge and downstream DOM. In Figure 1, the highlighted bars in the spectra for the three sampling locations show two narrower mass windows. Without any prior knowledge of the identities of the components giving rise to the various ions, simple visual comparisons between spectra offer insights into ions specific to the reservoir river outflow and sewage works DOM extracts. Figure 1(C.iii) clearly represents the effects of mixing of the two sources. Notable differences include the major ion at *m/z* 272.1642 present in the upstream DOM but absent from the sewage effluent. However, the downstream river DOM shows this ion at lower relative abundance due to the addition of compounds from the sewage outfall. In contrast, the dominant ion at *m/z* 274.2007 in the sewage works discharge spectrum remains the most abundant ion in the downstream extract despite dilution. All the other ions in the 4 *m/z* units mass window shown in Figure 1(iii) display similar behaviours relating to source specificity and dilution effects. However, it was quickly recognised that continuing with manual comparisons of this sort across the full spectral range would be prohibitively time‐consuming due to the many thousands of ions present in these mass spectra. Set out below is a new protocol for processing such a dataset to allow in‐depth interrogation of source contributions.

### 3.3 Statistical comparisons of DOM based on DI‐HRMS spectra

**Figure 2:**
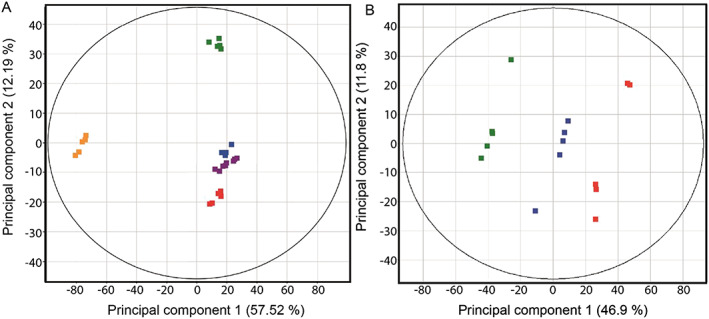
A, PCA of the DI‐HRMS spectra of the upstream (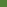), sewage outfall (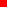), downstream (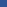), blank (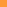) and mixed quality control (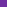) DOM extracts. B, PCA of the DI‐HRMS spectra of the upstream (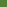), sewage outfall (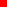), and downstream (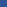) DOM extracts

The starting point for the statistical analyses is to establish if differences exist between the compositions of extracts in relation to the ions present and their intensities. The latter proceeds with creation of a data matrix of the ions aligned by their accurate masses and intensities for each DI‐HRMS spectrum. After this “peak picking” step the DI‐HRMS spectra were aligned to reveal 3237 ions detectable above a *s/n* ratio of 5. PCA was then applied to the generated data matrix to initially assess whether differences existed in composition between the extracts; the results are shown in Figure 2. In both PCAs the extracts clearly cluster in their respective replicate extraction groups. Figure 2A shows the mixed QC (purple) clusters between the downstream and sewage effluent replicate extracts, indicating that it is compositionally more similar to the latter extracts than to the upstream extract. The mixed QCs position on the PCA plot can be explained by the presence of the compounds contributed by the sewage outfall, but which are absent from the upstream extract. The DI‐HRMS spectra of the mixed QC, recorded every five extracts analysed throughout the analytical run, plot close together in the PCA, confirming that no major significant differences are attributable to analytical variance or data processing errors.

The PCA of the DOM extracts shown in Figure 2B highlights that there are distinct compositional differences between the upstream (green), sewage effluent (red), and downstream (blue) extracts; separation in principal component 1 (PC1) explains 46.9% of the total variance. The sewage outfall and upstream extracts are end members, confirmed by PC1 showing they are least similar in composition. As expected, the downstream extract plots between these groups, which is consistent with it being a mixture of the point source and reservoir riverine DOM.

**Figure 3:**
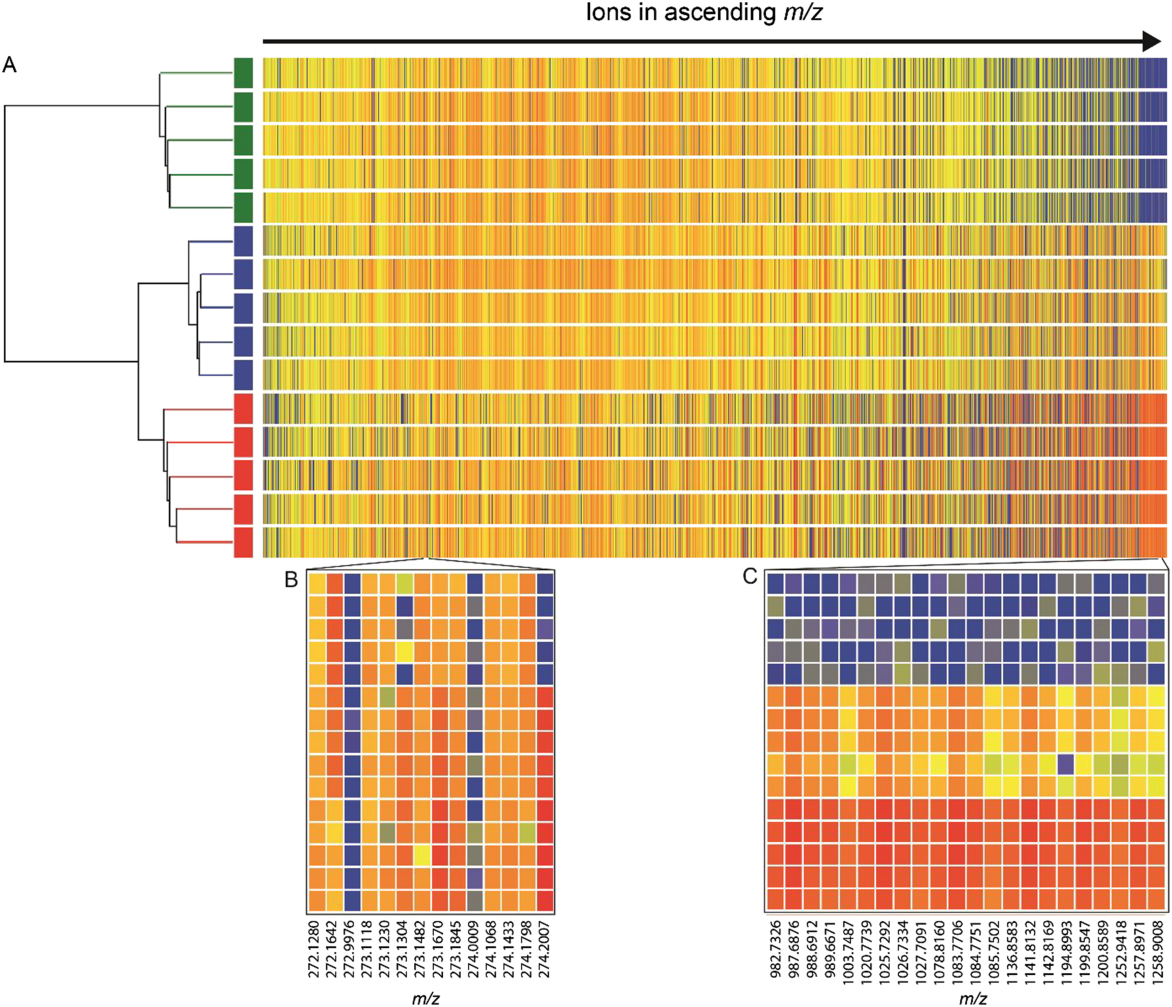
A, Hierarchical cluster analysis of the upstream (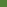), sewage outfall (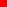), and downstream (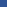) DOM extracts and heatmap of the ions detected in the DI‐HRMS. Comparison of the log_2_ of the intensity of the ions represented by colour with higher intensity hotter (red) and lower intensity colder (blue). Two narrower mass ranges B, *m/z* 272.0 to 274.5 and C, *m/z* 982.0 to 1259.0 from the heatmap

Hierarchical cluster analysis (Figure 3A) confirms that the upstream, downstream and sewage outfall extracts cluster in their respective replicate groups. However, the dendrogram also shows that, overall, the downstream and sewage outfall extracts are more similar in composition, as these separate further down the dendrogram than the downstream and upstream extracts. This further demonstrates the profound effect that the sewage outfall point source had on the downstream riverine DOM composition.

The heatmap visualises the differences in intensity of all the detected ions not easily determined when comparing DI‐HRMS spectra directly, and it shows ions changing in intensity across the mass range of the DI‐HRMS spectra. As discussed above when comparing the raw DI‐HRMS spectra directly (Figure 1), ions were present in the downstream and sewage outfall spectra of higher mass (*m/z* > 900), which were not seen in the upstream mass spectra; this can be clearly seen using the heatmap. Expanding the heatmap in this mass range shown in Figure 3C, the ions in this area of the heatmap are represented consistently in red indicating a high intensity in the sewage outfall extract, blue indicating low intensity in the upstream extract, and yellow/orange in the downstream extract, showing that the intensity falls between the upstream and sewage outfall extracts. This demonstrates the expected behaviour of compounds originating from the point source, i.e. that these are highest concentration in the sewage outfall, low concentration/absent upstream, and diluted upon entering the river in proportion to the river flow.

The ions in the mass range used in Figure 1(iii) are shown in the expanded heatmap in Figure 3B. The contrasting changes in intensity for *m/z* 272.1642 and *m/z* 274.2007 (discussed above) can also be seen in the heat map, occurring consistently across all extract replicates. In addition, using the heat map, more subtle changes can be seen, e.g. the ions *m/z* 273.1482 and 273.1670 exhibit the same high intensity in the sewage effluent and downstream extracts as shown by ion *m/z* 274.2007, which was not easily identifiable from directly comparing the DI‐HRMS spectra. This visualisation tool creates a quick approach to compare changes in the intensity of particular ions between the DI‐HRMS spectra and extraction replicates.

The upstream and sewage effluent DI‐HRMS spectra were compared using Kruskal‐Wallis analysis to highlight significant discriminating ions which differ between the mass spectra of the various extracts. A significance threshold p value <0.005 was chosen and only ions with a higher intensity in the sewage outfall than in the upstream spectra were retained, as these compounds were deemed most likely to derive from the sewage outfall. It was found that of the 3237 ions detected, 510 met these criteria; hence, these ions were selected for further analysis by MS/MS. The complexity of the DI‐HRMS spectra shows there are multiple ions within a 1 *m/z* unit mass range as illustrated by Figure 1(iii). Furthermore, each ion could represent multiple structural isomers. Isolation of precursor ions for further MS^2^ experiments from such a complex mixture would result in in chimeric product ion spectra, difficult to deconvolute and to match to reference spectra. This made it unfeasible even with the Orbitrap™ mass spectrometer to isolate a single ion from such a complex mixture.^59^ Therefore, HPLC/HRMS/MS was used to identify specific components.

### 3.4 HPLC/HRMS and HPLC/HRMS/MS analyses

**Figure 4:**
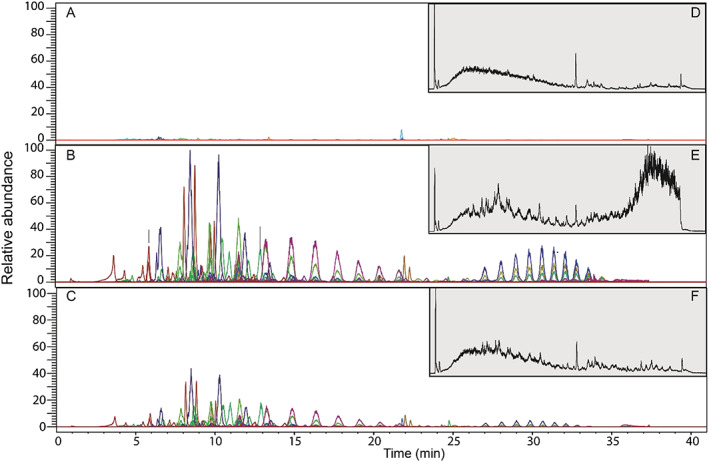
A, EIC of mass of the precursor ions identified in the upstream SPE extract; B, EIC of the precursor ions identified in the sewage effluent SPE extract; C, EIC of the precursor ions identified in the downstream SPE extract; D, TIC of upstream SPE extracts, E, TIC of the sewage outfall SPE extracts; and F. TIC of the downstream SPE extracts

The total ion chromatograms (TICs) of each of the SPE extracts shown in Figures 4D‐4F suggest poor chromatographic separation, because there are no individually resolved chromatographic peaks. However, plotting accurate mass extracted ion chromatograms (EICs) shows that individual compounds are separated chromatographically, and the apparently poor resolution actually arises from extensive co‐elutions inevitable in these extremely complex mixtures. Thus, HPLC/HRMS offers the following possibilities: (i) further exploration of extract composition and attribution of components to source, and (ii) isolation of individual ions allowing MS^2^ experiments to be carried out to identify compounds.

**Figure 5:**
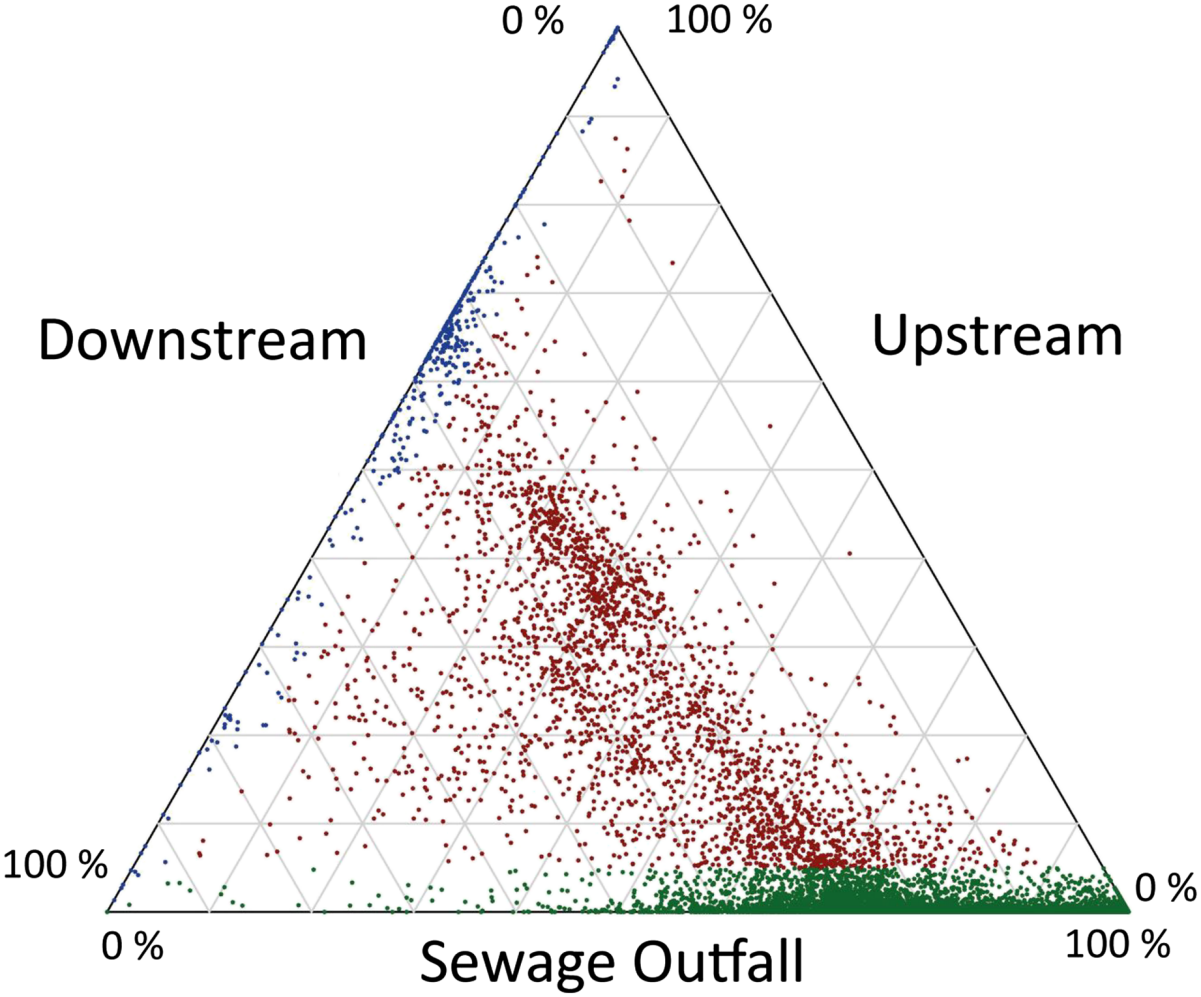
Ternary plot of ratios the peak areas of individual components detected in the SPE extracts. Green points highlight peaks where <5% of the component derives from the upstream contribution. Blue area highlights peaks where <5% of the component derives from sewage outfall. Red highlights peaks where >5% of the component can be attributed to all three sources

Even higher complexity is revealed through HPLC/HRMS than was apparent in the DI‐HRMS spectra. The “peak picking” algorithm detected 14,325 individual components across all extracts, which was recognised by aligning their unique masses and retention times (*m/z*@rt), producing a second data matrix of peak areas. A components peak area was compared in ratio form across the three different extracts using a ternary plot (Figure 5). The components found in each of the three extracts show three main trends. (i) The green area of the ternary plot highlights components where <5% of the total peak area is attributable to the upstream extract, confirming that these components derive from the sewage outfall and downstream extracts. As shown by the ternary plot most components have a higher contribution from the sewage outfall as these plot between 50 and 100% on the axis of the sewage outfall. This reflects their absence/low abundance in the river background (upstream), high abundance in the sewage effluent, and reduced abundance downstream due to in‐stream dilution. (ii) The blue area of the plot highlights components where <5% of the total peak area is attributable to the sewage outfall. This shows that these components are predominantly found in the river (downstream and upstream extracts). (iii) The red area highlights components where >5% of the peak areas is found in all three sources, showing that these components are common to all SPE extracts. The ternary plot facilitates the overall comparison of the different components detected in the HPLC/HRMS analysis, which is simply not possible through manual direct comparison.

Turning to the second use of HPLC/HRMS we focused on the 510 ions determined by DI‐HRMS as deriving uniquely from the sewage effluent. Using the accurate mass (± 5 ppm) 420 of these masses were detected as 681 components in the HPLC/HRMS spectra. This showed that a substantial proportion of the individual ions in the DI‐HRMS analysis represent more than one structure and that 90 of the masses were undetectable in the HPLC/HRMS spectra for a variety of reasons. The majority of the 681 components detected in HPLC/HRMS analysis plot in the green area of the ternary plot, confirming that these components derive from the sewage outfall.

These 681 components were compiled into a target list and analysed by HPLC/HRMS/MS as described above. The chromatographic separation allows the isolation of individual compounds for which product ion spectra can be recorded over multiple collision energies. Ninety‐six components were identified and the EICs of these are shown in Figures 4A‐4C. These EICs show that all 96 are present only in the downstream and sewage effluent extracts and none are detectable in the upstream extract, unequivocally confirming these compounds originate from the sewage treatment works. Interestingly, there is clearly a decrease in the peak area in the downstream extract compared with the sewage effluent extract, resulting from dilution of the point source by the river flow.

Of the 96 components identified, 72 related to the polymer PPG, alluded to above and discussed further below. The other 24 compounds were a mixture of pharmaceuticals, illicit drugs, flame retardants and metabolites, as summarised in Table 2. Twenty‐two of the compounds characterised have been previously identified in other sewage treatment effluents and/or surface water.^13^, ^15^, ^60^, ^61^ Two novel compounds were identified, namely the antiretroviral raltegravir and also piperine, which is a natural product derived from black pepper. The antiretroviral raltegravir was tentatively identified based on multiple CID spectra recorded at a range of energies. Further evidence for the identification of raltegravir was obtained using higher energy collision dissociation at the same collision energies used to obtain the reference spectra recorded in mzCloud (10–100 eV).

**Table 2:** Summary of the 24 identified compounds from the sewage effluent extract

Ion *(m/z)*	Retention time (min)	Fragmentation energy (eV)	p value	Formula	Compound	Product ions (*m/z*)
300.1592	3.67	40	0.0013	C_18_H_21_NO_3_	Codeine	282.1497, 267.1260, 253.1231, 243.1023, 225.0917, 215.1074, 199.0760, 193.0648, 187.0754, 183.0811, 175.0760, 165.0701, 161.0603
268.1544	3.93	30	0.0016	C_14_H_21_NO_4_	Atenolol acid	250.1441, 233.1176, 226.1079, 208.0971,191.0706, 165.0547, 145.0471, 116.1067, 98.0960
325.1915	5.47	30	0.0013	C_20_H_24_N_2_O_2_	Quinine	307.1782, 279.1521, 278.1570, 264.1315, 253.1296, 226.1199, 210.0940, 202.0851, 198.0880, 186.0918, 184.0739, 174.0926, 172.0744, 166.1228, 160.0798, 134.0914, 110.0951
290.1392	5.89	30	0.0013	C_16_H_19_NO_4_	Benzoylecgonine	272.1288, 168.1023, 150.0917, 124.1123, 122.0964, 119.0493, 91.0545
256.0152	5.9	40	0.0013	C_9_H_7_Cl_2_N_5_	Lamotrigine	229.0052, 221.0468, 220.0390, 213.9925, 210.9831, 193.0408, 186.9827, 185.9878, 183.9712, 179.0245, 173.9880, 171.9716, 166.0299, 165.0214, 158.9768, 151.0190
266.1657	6.07	40	0.0013	C_17_H_19_N_3_	Mirtazapine	235.1230, 223.1230, 209.1073, 195.0917
304.1549	7.04	40	0.0013	C_17_H_21_NO_4_	Cocaine	272.1281, 182.1176, 150.0913, 108.0807
253.0978	7.76	30	0.0013	C_15_H_12_N_2_O_2_	Carbamazepine 10,11‐epoxide	254.0817, 236.071, 210.09187,180.0809
278.2113	8.14	30	0.0013	C_17_H_27_NO_2_	Venlafaxine	261.206, 215.1435, 121.0641
373.1586	8.75	30	0.0013	C_20_H_24_N_2_O_3_S	Desacetyl diltiazem	373.1580, 328.1002, 223.0900, 178.0321, 150.04
260.1647	8.82	30	0.0013	C_16_H_21_NO_2_	Propranolol	242.1540, 218.1171, 183.0804, 157.0647, 132.1020, 116.1067, 98.0961, 86.0960
325.1711	9.68	30	0.0024	C_20_H_21_FN_2_O	Citalopram	325.1721, 307.1614, 280.1139, 262.1033, 234.0721, 166.0656, 156.0813, 116.0496, 109.0449
415.1456	10.06	30	0.0013	C_17_H_20_F_6_N_2_O_3_	Flecainide	415.1454, 398.1189, 386.12,370.0870, 332.1345, 330.05569,318.0558, 315.1075 301.0297
264.1752	11.15	30	0.0013	C_19_H_21_N	Nortriptyline	264.0840, 233.1331, 191.0860, 155.0861, 117.0700, 105.0700, 91.0543
278.1909	11.29	30	0.0013	C_20_H_23_N	Amitriptyline	278.1918, 233.1332, 191.0861, 179.0859, 155.0861, 117.0701, 105.0700, 91.0543
502.2957	11.53	30	0.0013	C_32_H_39_NO_4_	Fexofenadine	484.2830, 466.2726,262.1591, 250.5923, 246.1489, 233.1174,171.1168
237.1028	11.61	30	0.0013	C_15_H_12_N_2_O	Carbamazepine	237.0708, 220.0758, 194.0966, 192.0810
192.1388	13.54	30	0.0041	C_12_H_17_NO	*N,N*‐Diethyl‐3‐methylbenzamide, (DEET)	192.13829, 119.05, 100.07569, 91.0542
445.1636	13.21	30	0.0013	C_20_H_21_FN_6_O_5_	Raltegravir	361.1326, 318.1261, 278.0944, 253.0943, 236.0678, 193.07800 168.0780, 140.0824, 109.0451
286.1443	16.63	30	0.0013	C_17_H_19_NO_3_	Piperine	287.1490, 215.1071,201.0551, 173.0599, 150.0919, 135.0443, 112.0757
399.2512	21.99	40	0.0013	C_18_H_39_O_7_P	Tri(butoxyethyl) phosphate	299.1627, 243.1001, 225.0894, 199.0736, 143.0108, 124.0100, 101.0963, 98.9841
273.1855	22.32	30	0.0013	C_18_H_24_O_2_	Galaxolidone	255.1743, 227.1794, 203.107, 175.1117

As discussed above in relation to the DI‐HRMS spectra, 18 series of ions were highlighted differing by 58 *m/z* units in the downstream and sewage outfall extracts. The HPLC/HRMS TICs of these extracts showed no distinct series of chromatographically separated peaks with a normal distribution(s) which would be indicative of a synthetic polymer. However, using the accurate masses of the ions in each series (determined from DI‐HRMS) the EICs shown in Figures 4Band 4C, reveal two distinct normally distributed series of peaks, presumed to correspond to two series of oligomers. The first series elutes between 8 and 27 min with the most abundant oligomer eluting at 11.5 min and the second series elutes between 25 and 35 min with the most abundant oligomer eluting at 31 min. Oligomeric ions from five of the series were found to coincide with the earlier eluting distribution and three were found to coincide with the later eluting series. This indicates that these co‐eluting series are isotopes and adducts of the two different oligomer series.

The HPLC/HRMS/MS analyses of selected oligomeric precursor ions from both series produced product ions with a mass difference of 58.0419 *m/z* units, consistent with the cleavage of the ether bond in PPG. Using the accurate masses of the precursor and product ions it was possible to determine that the two series had different end groups. For the earlier eluting series, the end groups were determined to be dihydroxy, while the later eluting series possessed hydroxyl and butyl end groups. It was not possible to identify the remaining 10 series of ions with a mass difference of 58 *m/z* units from the DI‐HRMS spectra.

## 4 CONCLUSIONS

The results presented herein confirm the advantages of using an untargeted HRMS approach to the analysis of DOM contributed from point sources. The major findings of the research are:

- The DI‐HRMS molecular ‘fingerprints’ of the DOM extracts of river water obtained using SPE reveal the exceptional compositional complexity and very wide range of DOM compounds in waters which are not quantified, identified or controlled under current water quality legislation. The DI‐HRMS spectra of the DOM extracted from the upstream, downstream and sewage outfall water show how a point source can dramatically alter the composition of the riverine DOM.
- Manual assessments of the DOM composition, while revealing specific spectral features driving differences in DOM composition, emphasise the need to use chemometric statistical methods to interrogate datasets of this complexity.
- PCA of the DI‐HRMS spectra was readily able to resolve the different DOM sources, including in‐stream mixing. Hierarchical cluster analysis showed that the composition of the downstream DI‐HRMS spectra was more similar to that of the sewage outfall spectra than those of the upstream extracts, confirming the importance of the point source contribution to the overall DOM.
- Heatmapping facilitated visualisation of the changes in the intensity of ions between DI‐HRMS spectra including the determination of ion intensity changes which were not readily identifiable by directly comparing the DI‐HRMS spectra.
- Comparison of the sewage outfall and upstream DI‐HRMS spectra using Kruskal‐Wallis analysis provided a critical statistical data reduction step to identify the most important molecular species driving the differences in composition between the DOM extracts.
- HPLC/HRMS TICs show extensive co‐elution for the DOM extracts. However, the EICs of individual ions showed that compounds were separated chromatographically, with peak picking revealing over 14,325 components. Ternary plotting provided a visual means of attributing components to sources.
- A wide range of compounds was tentatively identified from the sewage outfall including pharmaceuticals, plasticisers, metabolites, and illicit drugs. Many have been identified in previous studies as originating from sewage treatment works. Others remain to be investigated to determine their environmental behaviour and potential ecosystem impact in waters.
- Industrially produced oligomeric PPGs were identified using DI‐HRMS and HPLC/HRMS in sewage effluent for the first‐time.

Overall, the results demonstrate that considerable value exists in combining DI‐HESI‐Orbitrap™‐HRMS and HPLC/HESI‐Orbitrap™‐HRMS for the analysis of complex DOM extracts. Our approach also highlights the value of applying statistical approaches to the assessment of complex data sets to determine the components differing between sources. Such an approach would have value in assessing compositional differences of any point source in river systems or between temporal events driven biologically, seasonally and/or anthropogenically.
